# Health effects of ambient air pollution – recent research development and contemporary methodological challenges

**DOI:** 10.1186/1476-069X-7-56

**Published:** 2008-11-06

**Authors:** Cizao Ren, Shilu Tong

**Affiliations:** 1 School of Public Health and Institute of Health and Biomedical Innovation, Queensland University of Technology, Kelvin Grove, QLD 4059, Australia; 2 Harvard School of Public Health, Exposure, Epidemiology and Risk Program, Landmark Center West, Suite 415, 401 Park Dr, Boston, MA 02215, USA

## Abstract

Exposure to high levels of air pollution can cause a variety of adverse health outcomes. Air quality in developed countries has been generally improved over the last three decades. However, many recent epidemiological studies have consistently shown positive associations between low-level exposure to air pollution and health outcomes. Thus, adverse health effects of air pollution, even at relatively low levels, remain a public concern. This paper aims to provide an overview of recent research development and contemporary methodological challenges in this field and to identify future research directions for air pollution epidemiological studies.

## Introduction

It is well known that exposure to high levels of air pollution can adversely affect human health. A number of air pollution catastrophes occurred in industrial countries between 1950s and 1970s, such as the London smog of 1952 [1]. Air quality in western countries has significantly improved since the 1970s. However, adverse health effects of exposure to relatively low level of air pollution remain a public concern, motivated largely by a number of recent epidemiological studies that have shown the positive associations between air pollution and health outcomes using sophisticated time-series and other designs [2].

This review highlights the key findings from major epidemiological study designs (including time-series, case-crossover, panel, cohort, and birth outcome studies) in estimating the associations of exposure to ambient air pollution with health outcomes over the last two decades, and identifies future research opportunities. We do not intend for this to be a formal systematic literature review or meta-analysis, but to discuss issues we feel are vitally important based on the recent literature and our own experience. This paper is divided into two parts: firstly to summarize recent findings from major epidemiological studies, and secondly to discuss key methodological challenges in this field and to identify research opportunities for future air pollution epidemiological studies.

## Health effects of ambient air pollution

### Time-series studies

There are a large number of time-series studies on the short-term health effects of air pollution, with the emphasis on mortality and hospital admissions by means of fitting Poisson regression models at a community level or ecological level. This type of time-series design is a major approach to estimating short-term health effects of air pollution in epidemiological studies for the last two decades. Many studies have found associations between daily changes in ambient particulate air pollution and increased cardiorespiratory hospital admissions [3-6], along with cardiorespiratory mortality [7-9] and all cause mortality [10]. Because numerous air pollution time-series studies show that exposure to air pollution is associated with different kinds of human health outcomes, it is impossible to list results from all studies. Table 1 only selects major time-series studies on short-term health effects of particulate matter (PM) and ozone from different countries around the world published over the last two decades because these two air pollutants are important toxic agents and widely explored by the majority of air pollution epidemiological studies. Early findings have been systematically and thoroughly reviewed by other authors [11,12].

**Table 1:** Time-series studies of short-term health effects of air pollution after 2000

**Study**	**Pollutant**	**Population**	**Methodology**	**Main findings**
Czech Republic and rural region in Germany [83]	TSP	Mortality 1982–1994	Poisson regression (GAM)	Czech Republic: 3.8% increase (95% CI: 0.8%, 9.6%) per 100 μg/m^3^; No evidence for association in the rural area in German at the Czech border.

10 US cities [84]	PM_10_	Mortality 1986–1993	Poisson regression (GAM)	0.67% increases for a 10 μg/m^3 ^(95% CI: 0.52%, 0.81%). No difference between summer and winter.

New Zealand [85]	PM_10_	Mortality Jun 1988–Dec 1993	Poisson regression (GAM)	1% increase for all-cause mortality (95% CI: 0.5%, 2.2%); 4% increase for respiratory diseases (95% CI: 1.5%, 5.9%)

10 US cities [21]	PM_10_	Mortality 1986–1993	Distributed lag model (GAM)	1.4% (95% CI: 1.15%, 1.68%) increase for 10 μg/m^3 ^on a single day using a quadratic distributed lag model; 1.3% increase (95% CI: 1.04%, 1.56%) using an unstrained lag model

20 US cities [2]	PM_10_, O_3_, SO_2_, CO, NO_2_	Mortality 1987–1994	Poisson regression (GAM)	PM_10_: 0.51% increase (95% CI: 0.07%, 0.93%) per 10 μg/m^3 ^for all causes; 0.68% increase per 10 μg/m^3 ^for cardiovascular and respiratory diseases (95% CI: 0.20%, 1.16%) O_3_: weaker evidence during the summer; Other pollutants: no evidence

Hong Kong [86]	PM_10_, SO2	Morality 1995–1998	Poisson regression (GAM)	Significant associations were found between mortalities for all respiratory diseases and ischaemic heart diseases (IDH). The increases for all respiratory mortalities (for a 10 μg/m^3 ^increase in the concentration) are 0.8% (95% CI: 0.1%, 1.4%) for PM_10 _and 1.5% (95% CI: 0.1%, 2.9%) for SO_2 _; the increases for IDH are 0.9% (95% CI: 0.0%, 1.8%) for O_3 _and 2.8% (95% CI: 1.2%, 4.4%) for SO_2_.

Seoul Korea[87]	PM_10_	Mortality 1995–1999	Poisson regression (GAM)	3.7% increase (95% CI: 2.1%, 5.4%) for non-accident causes, 13.9% increase (95% CI: 6.8%, 21.5%) for respiratory disease, 4.4% increase (95% CI: -1.0%, 9.0%) for cardiovascular disease and 6.3% increase (95% CI: 2.3%, 10.5%) for cerebrovascular disease per IQR increase of PM_10 _(43.12 μg/m^3^)

Shanghai, China [88]	PM_10_, SO_2_, NO_2_	Mortality Jun 2000 to Dec 2001	Poisson regression (GAM)	0.3% increase (95% CI: 0.1%, 0.5%) for PM_10_, 1.4% increase (95% CI: 0.8%, 2.0%) for SO_2 _and 1.5% increase (95% CI: 0.8%, 2.2%) for NO_2 _per 10 μg/m^3^

Brisbane, Australia [89]	BSP, O_3_, SO_2_, NO_2_	Hospital admission 1987–1994	Poisson regression (GLM)	BSP: 1.5% increase (95% CI: 0.6%, 2.3%) for respiratory diseases per 24-hr 10^-5^/m increase. O_3_: 2.3% increase (95% CI: 0.6%, 2.3%) for respiratory disease per 8-hr unit increase. SO_2_: 8.0% increase (95% CI: 3.0%, 13.1%) for respiratory disease per 24-hr unit increase. NO_2_: -0.1% increase (95% CI: -0.3%, 0.2%) for respiratory disease per 1-hr-max unit increase.

Brazil [90]	PM_10_, O_3_, SO_2_, CO, NO_2_	Respiratory disease Hospital admission 1993–1997	Distributed lag model	9.4% increase (95% CI: 7.9%, 10.9%) for 2 or less years old group and 7.0% (95% CI: 5.7%, 8.2%) for all age group per IQR PM_10 _increase (35 μg/m^3^); 1.6% increase (95% CI: 0.1%, 3.0%) for 2 or less years old group and 0.8% (95% CI: -7.5%, 9.2%) for all age group per IQR O_3 _increase (46 μg/m^3^); 5.9% increase (95% CI: 4.5%, 7.4%) for 2 or less years old group and 4.5% (95% CI: 3.3%, 5.8%) for all age group per IQR SO_2 _increase (14 μg/m^3^); 5.0% increase (95% CI: 3.3%, 6.8%) for 2 or less years old group and 4.9% (95% CI: 3.5%, 6.4%) for all age group per IQR CO increase (3 ppm); 9.4% increase (95% CI: 6.2%, 12.6%) for 2 or less years old group and 6.5% (95% CI: 3.3%, 9.7%) for all age group per IQR NO_2 _increase (80 μg/m^3^);

Single-site time-series studies have been criticized because of exposure measurement errors, substantial variation of the air pollution effects and the heterogeneity of the statistical approaches used in different studies [13]. Recently, several multi-site time-series studies have been conducted in Europe and the United States. Two large collaborative air pollution projects in Europe and U.S. are summarised below.

In Europe, the APHEA (Air Pollution and Health: a European Approach) studies have provided many new insights. Initial studies were based on older data (APHEA-1) [14] and a new series of studies (APHEA-2) used data of the PM_10 _fraction since the late 1990s [15]. The APHEA-2 mortality studies covered over 43 million people and 29 European cities, which were all studied for more than 5 years in the 1990s. The combined effect estimate showed that all-cause daily mortality increased by 0.6% (95% CI: 0.4%, 0.8%) for each 10 μg/m^3 ^increase in PM_10 _from data involving 21 cities. It was found that there was heterogeneity between cities with different levels of NO_2_. The estimated increase in daily mortality for an increase of 10 μg/m^3 ^in PM_10 _were 0.2% (95% CI: 0.0%, 0.4%), and 0.8% (95% CI: 0.7%, 0.9%) in cities with low and high average NO_2_, respectively [16]. The APHEA-2 hospital admission study involved 38 million people living in eight European cities. Hospital admissions for asthma and chronic obstructive pulmonary disease (COPD) increased by 1.0% (95% CI: 0.4%, 1.5%) per 10 μg/m^3 ^PM_10 _increment among people older than 65 years [15].

In the United States, the National Morbidity, Mortality and Air Pollution Studies (NMMAPS) focused on the 20 largest metropolitan areas in the USA, involving 50 million inhabitants, during 1987–94 [2]. All-cause mortality was increased by 0.5% (95% CI: 0.1%, 0.9%) for each increase of 10 μg/m^3 ^in PM_10_. The estimated increase in the relative rate of death from cardiovascular and respiratory disease was 0.7% (95% CI: 0.2%, 1.2%). Effects on hospital admissions were studied in ten cities with a combined population of 1 843 000 individuals older than 65 years [17]. The model used considered simultaneously the effects of PM_10 _up to the lag of 5 days and effects of PM_10 _on chronic obstructive pulmonary disease admissions to be 2.5% (95% CI: 1.8%, 3.3%) and on cardiovascular disease admissions to be 1.3% (95% CI: 1.0%, 1.5%) for an increase of 10 μg/m^3 ^in PM_10_. Bell et al. [18] analysed 95 NMMAPS community data to examine the association between ozone concentration and mortality, showing that a 10-ppb increase in the previous week's ozone was associated with a 0.5% (95% posterior interval (PI), 0.3%, 0.8%) increase in daily mortality and a 0.64% (95% PI, 0.31%, 0.98%) increase in cardiovascular and respiratory mortality. The effect estimates of the exposure over the previous week were larger than those considering only a single day's exposure. Recently, Dominici et al. [13] examined the short-term association between fine particulate air pollution and hospital admissions and found that exposure to PM_2.5 _was associated with different health outcomes. The largest association was observed for heart failure, and a 10 μg/m^3 ^increase in PM_2.5 _was found to be associated with a 1.3% (95% PI: 0.8%, 1.8%) increase in hospital admissions from heart failure on the same day.

Although time-series studies have shown that day-to-day variations in air pollutant concentrations are associated with daily deaths and hospital admissions, it is still unclear how many days, weeks or months of air pollution have brought such events forward. Harvesting or mortality/morbidity displacement means that some cases are occurring only in those to whom it would have happened in a few days anyway [19]. If so, the increase in cases immediately after exposure would be offset by a deficit in daily deaths a few days later [19,20]. If air pollution has harvesting effects, normal time-series models are unable to estimate the effects due to the issues of collinearity and statistical power. The polynomial distributed lag (PDL) model [21] and the time-scale model [19] have been adopted to explore whether air pollution has harvesting or displacement effects on daily deaths or hospital admissions. A few studies suggested potential harvesting effects of ambient air pollution while other studies have shown that there is no evidence for harvesting effects [19,22,23]. Although one study shows that potential bias might occur in PDL model [24], the estimated effects of ambient air pollutants seem to increase when longer lags of air pollution are included [19,20].

### Case-crossover studies

Case-crossover study design is an alternative approach to estimating short-term health effects of air pollution in epidemiological studies. In the last two decades, the case-crossover design has been applied in a large number of studies of air pollution and health [25-28]. For example, Neas et al. [27] used a case-crossover study design to estimate the association between air pollution and mortality in Philadelphia and found a 100 μg/m^3 ^increment in the 48 hours mean level of TSP was associated with increased all-cause mortality (odds ratio (OR) = 1.06; 95% CI: 1.03, 1.09). A similar association was observed for deaths in individuals over 65 years of age (OR: 1.07; 95% CI: 1.04, 1.11). Levy et al. [28] estimated the effect of short-term changes in exposure to particulate matter on the rate of sudden cardiac arrest. The cases were obtained from a previously conducted population-based case-control study and were combined with ambient air monitoring data. The results did not show any evidence of a short-term effect of particulate air pollution on the risk of sudden cardiac arrest in people without previously recognised heart disease. Schwartz [26] conducted a case-crossover study to examine the sensitivity of the association between ozone and mortality when adjusted for temperature and found that 10-ppb increase of maximum hourly ozone was associated with 0.23% (95% CI: 0.01% ~0.44%) increase in daily deaths after adjusting for temperature in 14 US cities. Barnett et al. [25] examined the association between air pollution and cardio-respiratory hospital admissions in Australia and New Zealand cities. The results show that air pollution arising from common emission sources was significantly associated with cardiovascular health outcomes in the elderly. For example, for a 0.9-ppm increase in CO, there were significant increases in elderly hospital admissions for 2.2% (95% CI: 0.9%, 3.4%) increase of total cardiovascular disease and 2.8% (95% CI: 1.3%, 4.4%) increase of all cardiac disease.

### Panel studies

Many air pollution panel studies have been conducted, including several large longitudinal studies of air pollution and health effects such as the Southern California Children's Health Study [29,30], in which children from grades 4, 7, and 10 residing in twelve communities near Los Angeles were followed annually. The results indicated that exposure to ambient particles, NO_2_, and inorganic acid vapour was associated with reduced lung function in children. Another large panel study, the Pollution Effects on Asthmatic Children in Europe (PEACE), was designed to examine the relationship between short-term changes in air pollution and lung function, respiratory symptoms and medication use [31]. This project was conducted in 14 centres using a common protocol in the winter of 1993–1994. Each PEACE centre involved an urban and a rural panel of symptomatic children and followed at least seventy-five 6–12 year old children [31]. The pooled estimates of two literature reviews which were separately conducted about the PEACE study and showed that no clear relation could be established for changes in PM_10_, black smoke, SO_2 _and NO_2 _and changes in respiratory health. The non-significant effects were thought to be possibly due to the short observation period. Ward and Ayres [32] reviewed 22 panel studies published in the 1990s to estimate the overall effects of ambient particles on children. Results show that the majority of identified panel studies indicated an adverse effect of particulate air pollution. Several recent panel studies also show that particulate air pollution is associated with human health [33-37].

### Cohort studies

Compared to time-series and case-crossover studies, there are only a few large cohort studies. About a dozen cohort studies have been conducted in the United States [38-44], Europe [45-48] and Australia [49]. A cohort study conducted by Dockery et al. [39] in six U.S. cities shows that there was a statistically significant and robust association between air pollution and mortality. The adjusted mortality rate ratio for the most polluted city was 1.26 (95% CI: 1.08–1.47) compared with the least polluted city. Air pollution was also associated with deaths from lung cancer and cardiopulmonary diseases. Abbey et al. [38] conducted a cohort study during 1973–1992 to estimate effect of exposure to long-term ambient concentrations of PM_10 _and other air pollutants, and show that PM_10 _was strongly associated with mortality from respiratory disease for both sexes adjusting for a wide range of potentially confounding factors. The relative risk (RR) for an interquartile range (IQR) difference of PM_10 _was 1.18 (95% CI: 1.42, 3.97). Ozone was strongly associated with lung cancer mortality for males for the IQR difference (RR: 4.19; 95% CI: 1.81, 9.69). Sulphur dioxide was also strongly associated with lung cancer mortality for both sexes. Pope et al. [44] conducted one cohort study in the US to examine the long-term effect of exposure to fine particulate air pollution. They found that fine particulate and sulphur oxide-related pollution were associated with all-cause, lung cancer and cardiopulmonary mortality. A 10 μg/m^3 ^increase in fine particulate air pollution was associated with an increase of 4%, 6%, and 8% for all-cause, cardiopulmonary, and lung cancer mortality, respectively. Hoek et al. [48] investigated a random sample of 5000 people and 489 of 4492 (11%) died during 1986–1994 in the Netherlands fining that cardiopulmonary mortality was associated with living near a major road with relative risk of 1.95 (95% CI: 1.09–3.52). A cohort study conducted by Filleul et al. [46] in France found that urban air pollution to be associated with increased mortality over 25 years in France. Frostad et al. [47], in a 30-year follow-up cohort study in Norway, found that respiratory symptoms were a significant predictor of mortality from all causes. In Australia, Jalaludin et al.[49] enrolled a cohort of primary school children with a history of wheeze (n = 148) in an 11-month longitudinal study to examine the association between ambient air pollution and respiratory morbidity. They found that PM_10 _and NO_2_, but not ozone, were significantly associated with doctor visits for asthma.

### Birth outcome studies

Even though effects of exposure to ambient air pollution on mortality and hospital admissions have been increasingly demonstrated over the past 30 years, exploring its adverse impact on pregnant outcomes has only begun since the last decade [50]. Because pregnancy is a period of human development particularly susceptible to the influence of many environmental factors due to high cell proliferation, organ develop and the changes of capabilities of fetal metabolism, the relative short-term period provides a unique opportunity to study the adverse effects of ambient toxins on human health [51]. The majority of birth outcome studies are based on large datasets routinely collected from air pollution monitoring systems and birth registration processes, and therefore, in general, the statistical power is strong [52-59]. Logistic regression models or linear regression models at the individual level are usually adopted to assess the effects of ambient air pollution on adverse birth outcomes adjusting for potential confounders including maternal age, maternal race, parity, fetal gender, season, gestational period, etc. Birth outcomes usually include low birth weight, preterm delivery and other biomarkers such as birth defect and ultrasound measures of head circumference. Personal exposures are often estimated at different terms, including the full gestation, trimesters, month after the pregnancy or before the time of delivery, etc.

Many studies have shown that there are significant associations between exposure to ambient air pollutants and adverse birth outcomes [52-60]. For example, Liu et al. [53] found that 5-ppb increase of sulfur dioxide was associated with an 11% (95% CI: 1%, 22%) increase of low birth weight (< 2500 grams) during the first month gestation and with a 9% increase of preterm delivery in Vancouver, Canada. A 1.0 ppm increase of carbon monoxide during the last month of pregnancy was associated with an 8% increase of preterm delivery. Parker et al. [60] selected population within 5 miles of over 40 air pollution monitoring sites across 28 California counties to estimate the adverse effects of air pollution and found that per 10 μg/m^3 ^PM increase was associated with 13 g (95% CI: 7.6 g, 18.3 g) decrease of birth weight. Similarly, Ritz et al. [59] conducted a population-nested case-control study to examine associations between air pollution and birth outcomes in Los Angeles and found that air pollution exposure was associated with preterm birth. Hansen et al. [58] examined the associations of exposure to ambient air pollution during early pregnancy with fetal ultrasonic measurements during mid-pregnancy in Australia. They found that a reduction in fetal abdominal circumference was associated with exposure to O_3 _during the days 31–60 of pregnancy (-1.42 mm, 95% CI: -2.74, -0.09), SO2 during the days 61–90 (-1.67 mm, 95% CI: -2.94, -0.40), and PM_10 _during the days 90–120 (-0.78 mm, 95% CI: -1.49, -0.08).

### Implications of weak health effects

Even though the association of air pollution with health outcomes is weak, it still has strong public health implications. One reason is that air pollution is ubiquitous and affects the whole population in most metropolitan cities. Another reason is that residents are continuously and permanently exposed to air pollution, which may have both short- and long-term effects on health outcomes. Some intervention studies have shown that the reduction in air pollution has resulted in an improvement in population health [55,61]. For example, Hedley et al. [61] reported that cardiovascular, respiratory and all cause mortality reduced by 2.0% (p < 0.05), 3.9% (p < 0.05) and 2.1% (p < 0.05) respectively in the first 12 months after an introduction of the restrictions on sulphur content of fuel in Hong Kong.

## Contemporary methodological challenges

Air pollution epidemiologic research is challenged by the complexity of human exposure to environmental agents and by the difficulty of accurately measuring exposure. Residents are usually ubiquitously exposed to air pollution. In order to detect small effects of air pollution, both high statistical power and sophisticated study design are required. In addition, the characteristics of air pollutants vary and their concentrations change both spatially and temporally. Although everyone is susceptible to high concentration of pollution, its concentrations are not evenly distributed across populations. Due to such complexities, there are still many research questions to be addressed by future air pollution epidemiological studies. The following section discusses these issues.

### Shape of exposure response curve

The shape of the exposure and response curve is very important. A key research question to be addressed is whether a threshold exists below which a certain air pollutant has no effect on population health. If such a threshold could be identified, public health benefits would be expected from bringing the pollutant below this level. Both theoretical and empirical works have been done to shed light on this issue [62,63]. In the analysis of NMMAPS data, no threshold evidence was found for the relationship between PM_10 _and daily all-cause and cardiorespiratory mortality [63]. By contrast, a threshold of about 50 μg/m^3 ^was indicated for non-cardiorespiratory causes of death – viz, below this point, PM_10 _had little influence on non-cardiorespiratory mortality. These issues remain to be clarified.

### Model uncertainty and bias

The process of model selection includes how to select covariates (eg, meteorological variables and co-pollutants), lag structure for air pollutants and the number of degrees of freedom for smoothing functions to adjust for long-term trend, short fluctuation, seasonality, other covariates and the determination of referent in case-crossover design. Studies have shown that the model choice will impact on estimates of relative risk [64]. As a result, many authors attempted to estimate the effects using the best single lag or combination of lags for meteorological factors and/or air pollutants and to identify the best degree of freedom for smoothing to adjust for different potential confounders. Some types of data can use several different models. Some authors do not clearly state why they select models and how they conduct data analyses. For example, when we estimate associations between exposure to air pollution and recurrent asthma episodes, based on different assumptions, at least five survival Cox models could be applied to estimate the associations between exposure to air pollution and asthma episodes [65]. Different assumptions or models may result in different estimates, and sometimes the difference is considerable. The choice of software options may cause this kind of uncertainty as well [65].

Results presented by the "best" final models are likely to cause publication bias because stronger and positive estimates tend to be published but negative results are usually difficult to be published. Multi-site time series design in which all data are analysed using the same model is one way to solve this problem. However, model uncertainty still exists in a multi-site study to some extent due to the model choice. Some studies have used Bayesian Model Averaging (BMA) to take into account uncertainties in model choice when making an inference [64]. BMA uses hierarchical models. The predictions and inferences are based on multiple models rather than a single model. Predictions are obtained by forming weighted averages of predictions over the different models where weights depend on the degree to which the data support each model.

Measurement errors of exposure to air pollution and potential confounders usually exist in air pollution epidemiological studies and it is impossible to be solved in most air pollution studies [66]. Due to spatial and temporal variations, data obtained in air pollution central monitors are not well representative of individual exposures. Some models are used to assess individual exposure to air pollution [66,67], but they could not efficiently adjust for measurement errors. Therefore, potential misclassification bias of exposure is one of the main concerns in air pollution studies.

There are both spatial and temporal variations for exposure and outcomes in air pollution studies [68]. Both times-series and case-crossover designs at a community level can efficiently adjust for some measured and unmeasured time-invariant characteristics of the subjects (such as gender, age, smoking status and spatial characteristics) via matching, and therefore, the potential confounding from these measured and unmeasured characteristics is minimised [69,70]. The key concern for these designs is how to control for temporal confounding and meteorological variables, such as seasonality, short-term variations and weather conditions (eg, temperature and humidity). In a prospective cohort study design, a major issue is how to identify a cohort with a sufficient variation in cumulative exposures, particularly when data recorded in central monitoring stations are used to measure ambient air pollution levels [44]. However, in maximizing the geographical variability of exposure the relative risk estimates from cohort studies are likely to be confounded by area-specific characteristics [68]. Due to collection of relatively detailed individual characters and sufficient adjustment for potential individual social and economic status, such confounding might be efficiently adjusted for.

Birth outcome studies are mainly based on routinely collected data, including exposure, outcome and potential confounders [52-60]. Most studies use pollution data obtained from the different monitors and the closest residential monitoring data are used as exposure proxies [58,60,71,72]. In general, information related to birth outcomes is well recorded in birth registration systems. However, the data may not include complete and accurate information on other potential confounders, such as maternal social and economic status and life styles. Birth outcome data analyses are usually conducted at an individual level. Therefore, this design is inherently vulnerable to some potential biases, including both temporal and spatial misclassification bias. Ritz and Wilhelm [73] has discussed the methodological issues of birth outcome designs in detail, and this review would not repeat these issues but rather than focus on potential bias in relation to spatial variation, which was ignored in their review, in the following section.

In birth outcome studies, both exposure and outcome data include temporal and spatial variations to some extent. The majority of birth outcome studies have adjusted for temporal and other confounders which are related to delivery information, including season, maternal age and race, fetal gender, parity, and maternal education attainment [52-60,71,72]. However, so far, few studies have paid much attention to the potential spatial confounding. Unlike time-series or case-crossover studies, most birth outcome studies lack the ability for automatic adjustment for measured and unmeasured time-invariant spatial variations. Unlike cohort studies, birth outcome study designs also lack the ability to efficiently adjust for personal life styles and social and economic status due to the lack of the detailed information available in routinely collected data. Because both exposure to air pollution and birth outcomes are influenced by some geographic characteristics, such as land use, forest, public infrastructure, and residential social and economic status, etc, the previous birth outcome studies might introduce bias to some extent due to the failure to consider spatial variation. In general, these spatial-related factors are favourable to links between air pollution exposure and birth outcomes. Therefore, we presume that the stronger associations reported in the previous birth outcome studies might partially attribute to this kind of bias. The simple way to adjust for the spatial variation is to add a categorical variable for individual residential areas to fit a fixed effect model or to include the residential areas to fit a mixed model or a random effect model.

### Interaction of temperature and air pollution

In many locations, patterns of air pollution are driven by weather. Therefore, concentrations of air pollutants may be associated with temperature. Therefore, it may be possible that temperature and air pollution interact to affect health outcomes. Although effect modification has important public health implications [74], this issue has so far received limited attention, probably because of methodological complexity and the difficulty in data interpretation. Several studies examined whether or not ambient air pollution and temperature interact to affect human health outcomes, but they produced conflicting results [75-78]). For example, Samet et al. [78] investigated the sensitivity of the particulate air pollution mortality effect estimate to alternative methods of controlling weather and did not find any evidence that weather conditions modified the associations of particulate air pollution and sulphur dioxide with mortality, regardless of approaches of synoptic weather conditions. Katsouyanni et al. [75] used a multiple linear regression to investigate the interaction between air pollution and high temperature during a heat wave in Athens in July 1987. They found that while the main effects of air pollution index were not statistically significant, there was statistically significant synergistic effect between high levels of sulphur dioxide and high temperature (P < 0.05). Roberts [77] found evidence that the effect of particulate air pollution on mortality might depend on temperature but the synergistic effect was sensitive to the number of degrees of freedom used in confounder adjustments. Recently, we found that temperature and particulate matter symmetrically enhanced the effect [76]. Since then, several multiple-site studies have found evidence that temperature and air pollutants interacted to impact human health but the nature and magnitude of such an interaction varied with geographic area [79-82]. Thus, further research is needed to examine the interactive effects between air pollutants and temperature on mortality and morbidity, especially in different spatial settings.

## Conclusion

Many time-series, case-crossover and panel studies have shown that there are consistent short-term effects of air pollution on health outcomes (hospital admissions or deaths). Some cohort studies have also shown long-term health effects of air pollution. In spite of the weak associations of air pollution with human morbidity or mortality, its public health implications are strong because exposure to air pollution is ubiquitous and widespread. However, there are several key methodological challenges in the estimation of the health effects of low-level exposure to air pollution, such as the shape of the exposure response curve, threshold of air pollution, interactive effects of air pollution and weather conditions, and model uncertainty and potential bias. Future research efforts should focus on these important issues.

## Competing interests

The authors declare that they have no competing interests.

## Authors' contributions

CR conceived of the study, participated in its design, and is responsible for the draft of the manuscript. ST participated in the study design and revised the draft of the manuscript.
